# The Newly Normed SKT Reveals Differences in Neuropsychological Profiles of Patients with MCI, Mild Dementia and Depression

**DOI:** 10.3390/diagnostics9040163

**Published:** 2019-10-25

**Authors:** Hartmut Lehfeld, Mark Stemmler

**Affiliations:** 1 Department of Psychiatry and Psychotherapy, Paracelsus Medical University, 90419 Nuremberg, Germany; 2 Institute of Psychology, University of Erlangen-Nuremberg, 91052 Erlangen, Germany; mark.stemmler@fau.de

**Keywords:** differential diagnosis depression vs. MCI/dementia, mild cognitive impairment, dementia, depression in old age, SKT (Syndrom-Kurztest), cognitive assessment

## Abstract

The SKT (Syndrom-Kurztest) is a short cognitive performance test assessing deficits of memory and attention in the sense of speed of information processing. The new standardization of the SKT (2015) aimed at improving its sensitivity for early cognitive decline due to dementia in subjects aged 60 or older. The goal of this article is to demonstrate how the neuropsychological test profile of the SKT can be used to provide valuable information for a differential diagnosis between MCI (mild cognitive impairment), dementia and depression. *n* = 549 patients attending a memory clinic (Nuremberg, Germany) were diagnosed according to ICD-10 and tested with the SKT. The SKT consists of nine subtests, three for the assessment of memory and six for measuring attention in the sense of speed of information processing. The result of the SKT test procedure is a total score, which indicates the severity of overall cognitive impairment. Besides the summary score, two subscores for memory and attention can be interpreted. Using the level of depression as a covariate, statistical comparisons of SKT test profiles between the three patient groups revealed that depressed patients showed more pronounced deficits than MCI patients in all six attention subtests. On the other hand, MCI patients displayed significantly greater mnestic impairment than the depressed group, which was indicated by significant differences in the memory subscore. MCI and dementia patients showed similar deficit patterns dominated by impairment of memory (delayed recall) with MCI patients demonstrating less overall impairment. In sum, the SKT neuropsychological test profiles provided indicators for a differential diagnosis between MCI and beginning dementia vs. depression.

## 1. Introduction

Dementia and depression are the most frequent psychiatric disorders of old age [1]. Both affect quality of life of patients in a more fundamental way and to a much greater extent than many somatic diseases [2]. Depression is also considered a serious risk factor for developing dementia [3,4]. In addition, dementia and depression share a diagnostic deficit. Dementia is often only diagnosed in more advanced stages showing higher degrees of functional impairment [5]. Worldwide, patients suffering from depression frequently are not correctly diagnosed; therefore, in many countries less than 10% of depressed subjects receive adequate treatment [6].

Due to an overlap in symptoms, a valid differential diagnosis between dementia and depression is sometimes difficult to establish: Depressive disorders in old age are associated with cognitive impairment in 40% to 60% of patients [7]. Conversely, about 40% of dementia patients develop depression symptoms [8,9]. Accordingly, among the differential diagnoses of dementia, in the first place the ICD-10 [10] lists depressive disorders, which can show characteristics of incipient dementia with memory impairment, slowed thinking and lack of spontaneity. In the same way, the DSM-5 [11] recommends inspecting the cognitive profiles of patients suggesting memory and executive impairment as typical for Alzheimer’s disease, whereas nonspecific and more variable test performance could be expected in major depression. In accordance with this perspective, a number of reviews state a lack of clarity in the neuropsychological profiles of depressive disorders [12,13]. However, other authors consider impairment in speed of information processing, attention or executive functions as cognitive core features of depressed older patients [7,14,15,16].

Since the cognitive deficits associated with depression are less pronounced than those found in dementia [17,18,19], making a differential diagnosis much more difficult when it is not a full-blown dementia, but “mild cognitive impairment” (MCI), which has to be differentiated from depression. For almost 30 years, MCI has been conceptualized as a transitional phase between normal aging and dementia; it is discussed as a clinical condition with a high prognostic value for future dementia development, mostly towards Alzheimer’s dementia [20,21,22]. The diagnostic differentiation of MCI and depression is further complicated by the existence of several MCI subtypes (amnestic vs. non-amnestic, single vs. multiple domains) causing a potential variety of neuropsychological performance patterns. Furthermore, nearly one third of MCI patients also will develop depression symptoms [23]. Overall, a wide range of disturbed cognitive functions may be expected in both MCI and depressed subjects. Consequently, attempts to differentiate between MCI and depression by means of psychometric tests often have failed [18,19,24].

Against this background, the present study compared the neuropsychological profiles of patients with MCI, mild dementia and depression tested with the SKT according to Erzigkeit [25]. The SKT (acronym for Syndrom-Kurztest; however, this German term is outdated and not used anymore) is a short cognitive performance test assessing memory and attention, the latter in the sense of speed of information processing. Thus, the SKT addresses exactly those two cognitive domains that are considered to be primarily impaired in patients with mild dementia and depressive disorders, respectively. Furthermore, given the fact that amnestic MCI is the most frequent MCI subtype [24,26], it was expected that patients with MCI or mild dementia would show greater deficits in the memory section of the SKT, while depressed patients would be more impaired in subtests measuring speed of information processing.

## 2. Methods

### 2.1. Samples

The present study included all patients referred between 2000 and 2005 to the Memory Clinic of Nuremberg General Hospital fulfilling the following criteria: (1) age 60 years or older, (2) diagnosis of mild cognitive impairment (MCI, in accordance with the consensus criteria according to Winblad et al. 2004 [20]), mild dementia (Alzheimer type, mixed type or vascular dementia; ICD-10 codes F00 or F01) or depressive disorder (ICD-10 codes F32 or F33) and (3) complete assessment with all SKT subtests. As an indicator of the clinical severity of MCI and mild dementia, assignment to stages 3 (MCI) or 4 (mild dementia) of the Global Deterioration Scale (GDS, [27]) was required. Exclusion criteria were (1) age below 60 years, (2) all other diagnoses than the ones required for inclusion, e.g., other forms of dementia (dementia in Parkinson disease and amnesic syndromes due to substance use), other forms of depression (e.g., adjustment disorders or post-traumatic stress disorders) and (3) not being able to complete all SKT subtests (e.g., due to reduced motor abilities, due to not being able to understand the test instructions or being unfamiliar with numbers).

### 2.2. Measures

**Figure 1:**
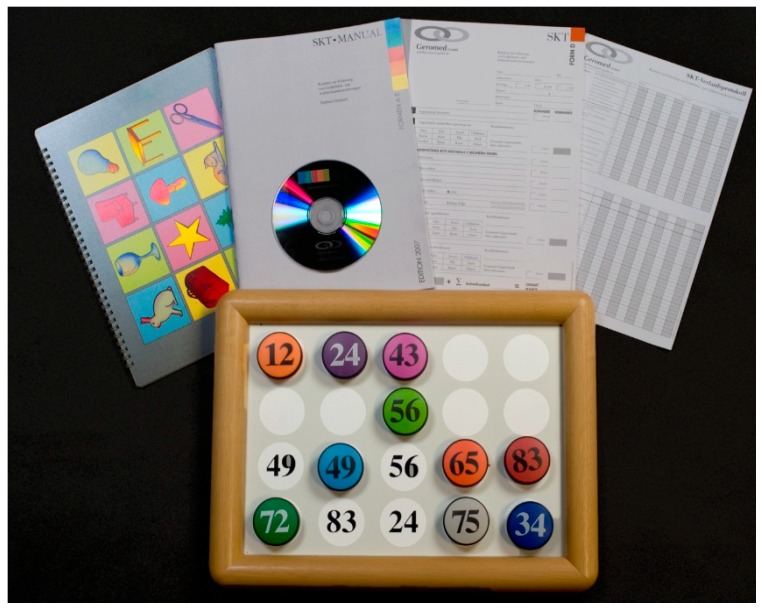
SKT test materials.

The SKT is a cognitive test developed and published in Germany [28] assessing impairment of memory and attention, the latter in the sense of speed of information processing. The SKT comprises nine subtests, three of which refer to visual memory (immediate and delayed recall and recognition memory), the remaining six subtests measure processing speed. An overview of the subtests and the tasks to be completed is given in Table 1, the test materials are shown in Figure 1.

The maximum performance time for each subtest is limited to 60 s, so that the total administration time will be approximately 10 to 15 min. In the attention/speed subtests, the patient is instructed to work as fast and accurately as possible. In the memory subtests, all correct answers given within 60 s will be scored. The test was developed in five parallel forms (A to E) for repeated test administration even within short time intervals. In addition to a total summary score, the evaluation also provides subscores for separately interpreting memory and attention performance.

Since its publication in 1977, the SKT has been revised three times. The last revision carried out in 2015 was undertaken to establish new test norms for age groups 60 years and older to improve the sensitivity of the SKT for early cognitive decline due to Alzheimer’s disease or other neurocognitive disorders [25]. In a first step, more than 1000 non-demented community dwelling subjects aged between 60 and 91 years were tested with the SKT. On the basis of this data set, conditional expected values were calculated for each of the nine SKT subtests using multiple regressions taking into account age, gender and level of intelligence. Based on the deviations from the predicted performance, in a second step, norm scores of 0, 1 or 2 were defined depending on the size of the deviation of the actual performance from the predicted performance (higher scores indicating greater cognitive impairment). The SKT total summary score ranging between 0 and 18 is obtained by adding the deviation scores (i.e., norm scores) of the nine subtests and is visualized in a traffic light system. Total scores between 0 and 4 indicate “age-appropriate cognitive performance” (green), scores between 5 and 10 points suggest “mild cognitive impairment” (MCI, yellow) and values between 11 and 18 substantiate a “suspicion of beginning dementia” (red). It must be noted that the SKT total summary score can be reliably interpreted in case of a homogeneous test profile, i.e., the memory and attention domain are affected to a similar extent. In case of profile heterogeneity, the summary scores should be interpreted with caution and the severity of impairment should also be assessed separately for the two domains.

Besides the SKT, the standard test battery of the Nuremberg Memory Clinic comprised the CERAD-NP [29] and two different depression scales [30,31]. Furthermore, relatives rated the patient using the Bayer-ADL scale to stage functional capacities [32] and the Neuropsychiatric Interview [33] to assess behavioral disturbances occurring in dementia. The diagnostic classification of a given patient was made taking into account information from different sources (anamnesis, medical examination, neuropsychology, everyday functioning, and neuroimaging or laboratory results).

### 2.3. Statistics

The raw scores of the nine SKT subtests were converted into norm scores using the EXCEL program “SKT-Analyser-v10.xlsm” [25]. From these, the subscores for memory and attention as well as the SKT total summary score were calculated by adding the corresponding subtest scores (SKT subscores and total summary score are also included in the program printout). Using one-way analyses of variance, differences in the nine subtests, the two subscores and the SKT total summary score were checked for statistical significance between the three study groups. Pairwise group comparisons were based on the Tukey test. A Bonferroni correction for multiple comparisons was not carried out, as the focus of the analyses presented here was on the comparative examination of test profiles and less on the detection of robust group differences. Comparisons of SKT profiles across the nine subtests and the memory and attention subscores were performed using multivariate analyses of variance for repeated measurements. To assess the effect of depression on SKT scores, Pearson correlation coefficients were computed between depression scores [30,31] and the SKT summary score, the SKT memory and attention subscores and the norm scores of the nine SKT subtests. Moreover, we repeated the analyses of variance controlling for depression to establish a “pure” metric of cognitive impairment unbiased by affective disturbances. While we used two different depression scores, we calculated the mean of the transformed z-scores [30,31]. Furthermore, a receiver operator characteristics (ROC) analysis was employed to compute areas under the curve (AUC) for each of the three diagnostic groups using the SKT norming sample comprising 1053 non-demented community dwelling subjects aged between 60 and 91 as a reference group. All analyses were carried out with the statistics program IBM SPSS Statistics (Version 20, Armonk, NY, United States) and were based on a completely anonymized data set. The study was registered in the study centre of the Nuremberg General Hospital as a quality assurance measure according to § 27/4 of the Bavarian Hospital Law.

## 3. Results

**Figure 2:**
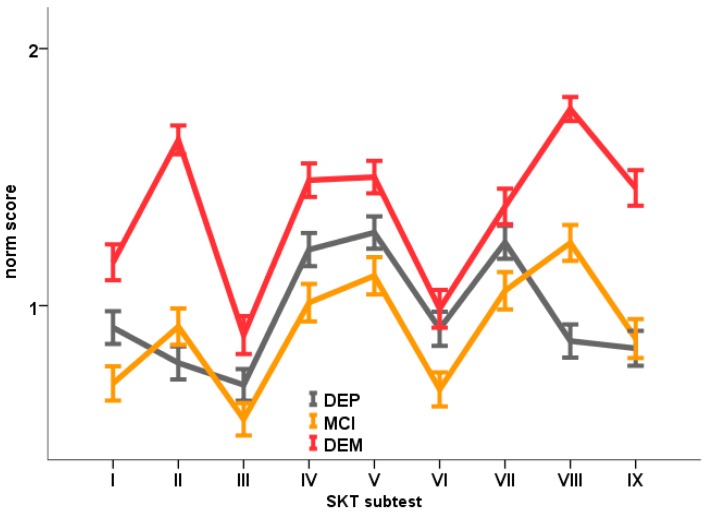
Norm scores (means) of the three study groups for the nine SKT subtests (I: naming objects, II: immediate recall, III: naming numerals, IV: arranging blocks, V: replacing blocks, VI: counting symbols, VII: reversal naming, VIII: delayed recall, IX: recognition memory).

Of the 1362 patients assessed between 2000 and 2005 in the Nuremberg Memory Clinic, a total sample of *n* = 549 fulfilled the inclusion and exclusion criteria (see Section 2.1). The patients were distributed among the three diagnostic groups as follows: 172 patients were diagnosed with MCI, 166 patients were diagnosed with dementia (F00.0 or F00.1: 89 patients, F00.2: 39 patients and F01: 38 patients), 211 patients suffered from first manifested or recurrent depression (F32: 150 patients and F33: 61 patients). Diagnoses were based on ICD-10 [10]. Sociodemographic data and SKT results (subtests, subscores and SKT total score) of the three study groups were compiled together with the results of the group comparisons in Table 2. The SKT test profiles of the three study samples are depicted in Figure 2.

As Figure 2 illustrates, the MCI and dementia group show peaks in their SKT profiles in subtest VIII, which examines the delayed recall of objects. In contrast, depressed patients reveal their most striking performance deficits in the speed subtests IV, V and VII. Furthermore, Figure 2 indicates less overall cognitive impairment in MCI and depressed subjects when compared to dementia patients. SKT total summary scores for the MCI and depressed groups displayed values between 8 and 9 points; they do not differ statistically between both groups (see Table 2). However, striking differences can be detected in their subtest profiles. While mean scores in memory subtests II, VIII and IX of MCI patients are consistently lower than those of subjects with dementia (level significance was reached for subtest VIII), depressed patients show more pronounced deficits than MCI patients in all six speed tests (with only the difference in subtest VI turned out to be significant, statistical tendencies (*p* < 0.10) were found for subtests I and IV). Subsequently, the memory subscore indicated significantly greater cognitive impairment in the MCI group and the attention subscore in the depression group (*p* < 0.05 each).

The comparison of the SKT profiles between the three diagnostic groups included in the study across all nine subtests revealed a highly significant interaction effect ‘diagnosis × subtest’ (Pillai’s Trace = 0.121 with F (16, 1080) = 4.34, *p* < 0.000) in a multivariate analysis of variance with repeated measures (MANOVA), which indicates an overall difference of test profiles. Subsequent pairwise comparisons performed to identify the source of this interaction effect revealed a marginally non-significant interaction (Pillai’s Trace = 0.045 with F (8, 329) = 1.59, p = 0.054) for the comparison MCI vs. dementia, indicating a relative similarity of the subtest profiles between these two groups. The two remaining contrasts, MCI vs. depression and dementia vs. depression, were again significant with respect to the interaction term ‘diagnosis × subtest’ (MCI vs. depression: Pillai’s Trace = 0.066; F (8, 329) = 3.31, p < 0.001; depression vs. dementia: Pillai’s Trace = 0.0148; F (8, 329) = 8.01, p < 0.000) pointing towards the depression group as the source of the overall difference between profiles.

More clearly than the profile comparisons across subtests, the comparison of the SKT memory and attention subscores revealed the different impairment patterns between diagnostic groups MCI/mild dementia vs. depression. When comparing the three subsamples, the interaction ‘diagnosis × subscore’ reached significance (Pillai’s Trace = 0.034; F (2, 546) = 2.55, p < 0.000). However, when comparing only MCI vs. dementia, the level of significance was missed more clearly for the SKT subscore profile than for the subtest profile (Pillai’s Trace = 0.004 with F (1, 336) = 1.18, p = 0.277). This result demonstrates the similarity of the SKT subscore profiles between MCI and mild dementia. The remaining comparisons (MCI vs. depression and dementia vs. depression) again showed significant interaction effects, which can be interpreted in terms of different subtest compositions in MCI/mild dementia vs. depression (MCI vs. depression: Pillai’s Trace = 0.042; F (1, 381) = 16.52, p < 0.000; dementia vs. depression: Pillai’s Trace = 0.024; F (1, 375) = 9.18, p < 0.01).

Pearson correlation coefficients between SKT and depression scores ranged between r = −0.15–0.20 in the total sample and hardly exceeded r = 0.20 in the three subsamples (MCI: range *r* = 0.02–0.20; DEM: range *r* = −0.22–0.15; DEP: range *r* = −0.03–0.17). Accordingly, introducing depression as a covariate into the analyses of variance did not fundamentally change the outcome. Regarding significance, seven out of eight comparisons remained significant, even though less pronounced. Noteworthy, the differences in SKT subtest and subscore profiles between MCI vs. depression outlasted the correction for depression. When comparing these two groups, the interaction terms remained significant (diagnosis × subtest: Pillai’s Trace = 0.046; F (8,351) = 2.130, p = 0.033; diagnosis × subscore: Pillai’s Trace = 0.017; F (1,358) = 6.29, p = 0.013). Finally, Table 3 displays the results of the ROC analyses examining the ability of the SKT sum score and the subscores to correctly classify MCI, dementia and depression. All SKT scores were based on the SKT norming sample used for developing the regression based norms [25].

## 4. Discussion

In the present analyses, the newly-normed SKT, a short cognitive performance test for assessing deficits of memory and attention, revealed different neuropsychological profiles for patients belonging to the MCI/mild dementia spectrum on the one hand, and patients suffering from depressive disorders on the other. In the MCI and dementia conditions, the deficit patterns displayed their peaks for the delayed memory recall of objects. Since amnestic MCI (isolated or in combination with other cognitive domains) is considered to be the most frequent MCI subtype [24,26] and impaired episodic memory is a prerequisite for a dementia diagnosis according to the ICD-10 criteria, this result is not really surprising. However, it can be taken as an indication of the construct validity of the SKT as a tool to support diagnosis in organic mental disorders. It may be expected that the assessment of patients with other forms of dementia, e.g., Lewy-Body, Frontal Lobe or Parkinson’s, might have resulted in different test profiles. In the same vein, an exploratory investigation comparing SKT subtest patterns of patients diagnosed with Alzheimer’s and Parkinson’s dementia using the old test norms [34] indicated greater impairment of Parkinson patients in subtests assessing speed of information processing with subtest V (replacing blocks) reaching the level of statistical significance. Moreover, the slowing of speed of information processing, especially in tasks with a strong executive component, which could be observed in the depressed sample of the study has been described as a characteristic neuropsychological feature of depression [7,14,15].

To address a common misunderstanding, it must be pointed out that the SKT is not a test exclusively for the area of dementia. Originally, it was developed for usage with patients older than 17 years of age suffering from acute or chronic mental disorders irrespective of their aetiology. Therefore, there is ample experience with the SKT in the cross-sectional and longitudinal assessment of cognitive impairment resulting, e.g., from brain injury, substance abuse or anesthesia [35]. The misclassification of the SKT as a dementia test was surely supported by the fact that the SKT has been used as an outcome measure in more than 50 studies investigating the efficacy of various nootropic compounds, cognition enhancers or antidementia drugs, in the past years with a clear focus on the efficacy of Ginkgo biloba [35].

In line with this shift of test usage towards dementing disorders starting in the 1980s, all three test revisions of the SKT focused on older patients suffering from cognitive impairment. The first modification in 1989 aimed at making test materials more appealing [36]. The second revision suggested a finer classification of age norms beyond the age of 65 and included an option for separate assessment of memory and speed functions allowing for differential diagnostic considerations [37]. Finally, the new norming of 2015 [25] served the purpose of improving the sensitivity of the test for early recognition of dementia in persons aged 60 years or older. First data show the high sensitivity and specificity of the SKT for dementia being 0.83 and 0.84, respectively [38,39]. The results of the ROC analyses reported in the present study support these findings.

Of special interest in the present investigation is the finding that the analysis of the SKT subscores for memory and attention revealed statistically significant differences between MCI and depressed patients. Other working groups, e.g., Barth et al. (2005) [18] using the CERAD-NP test battery did not find significant differences between MCI and depression in any of the CERAD tasks. In the same way, Zihl et al. (2010) [24] analyzing neuropsychological test data of MCI and cognitively impaired depressed patients also applying the CERAD-NP and an additional series of other psychometric instruments did not receive a single significant difference between both diagnostic groups. Nevertheless, they identified a significant reduction in speed of information processing for their depressed patients when comparing the results to cognitively normal older controls. This may be taken as a further indication that processing speed is a core domain affected by depression, which is in full accordance with the present results. Accordingly, our ROC analyses for depression vs. controls revealed a higher discriminative power of the SKT speed subscore in comparison to the memory subscore. Furthermore, the fact that the differences in SKT subscores for memory and speed performance outlasted a correction for (self-rated) depression may cautiously be considered as a hint of reduced speed of information processing as a trait marker for depression. This interpretation is supported by results that speed and executive test performance of depressed patients who were successfully treated was improved, but not normalized [15]. Finally, in a study by Dierckx et al. (2007) [19] a cued recall paradigm discriminated well between Alzheimer patients and depressed subjects, but considerably lost diagnostic accuracy for separating MCI from depression. The authors explain this finding by the heterogeneity among MCI patients and a diagnostic uncertainty induced by misdiagnosing MCI in the presence of affective symptoms as depression.

Differential diagnosis between MCI/dementia and depression is not only complicated by an overlap in cognitive and affective symptoms. Meanwhile, there is evidence that MCI/dementia and depression share common pathophysiological pathways (e.g., [40,41]). On the one hand, depression seems to play a role in the pathogenesis of Alzheimer’s disease via stress and a glucocorticoid increase that may cause amyloid-beta production or hippocampal atrophy resulting in an elevated dementia risk in depressed subjects. On the other hand, neurodegenerative and cerebrovascular alterations in the brain are discussed as etiological factors of depression [42,43]. Thus, in the future it is desirable that the clinical and psychometric assessment of patients suffering from cognitive and/or affective symptoms should be supplemented by information available from biomarkers reflecting neuronal or vascular damage. This could allow for defining MCI [21] and depression subgroups bearing a higher risk for cognitive decline towards a dementia syndrome. The next step for our working group will be an analysis of SKT follow-up data that might be available for MCI and depressed patients participating in the present investigation to validate their diagnostic classifications.

A final remark refers to the international validity of the SKT, which up to about 1990 was mainly used in German-speaking countries. However, in the following years an increasing number of international studies were performed, e.g., in the United States, the UK, Greece, Russia, Chile, Mexico, Brazil or South Korea [44,45,46,47,48,49,50]. Some of these studies specifically aimed at validating the SKT for the respective target language or culture. To summarize a few findings, the transcultural transfer of the (mostly nonverbal) SKT test materials only required minor adjustments of some objects shown in subtest I (because they were less familiar in the target countries) or the adaptation of letters to be read in subtest VII (especially for countries using non-Latin letters). In many of these studies, the SKT kept the psychometric properties or factor structure comparable to the original German test version. However, the dependency of test results on education becomes critical especially with patients from developing countries with very few years of formal school education [47,49].

In 2019, the German standardization study of 2015, which established the new testing norms was replicated in three testing centers in the USA, Australia and Ireland with a somewhat smaller sample of altogether 285 cognitively unimpaired persons aged between 60 and 96 years [35,51]. As in the German study, the most important predictors of the SKT performance were age, age-squared, gender and intelligence. The explained variance was comparable to that found in the German standardization sample suggesting that the regression-based German SKT norms from 2015 are well matched by those found in 2019 for English speaking subjects. This equivalence may be taken as evidence for the cross-cultural stability of the SKT in German and English speaking countries of the Western world (see also [45]). It indicates that the SKT in its present form may be used without any further adaptations of the testing material in these regions. Taken together, the results of the present study confirm that the SKT can be considered as a neuropsychological test instrument validly assessing impairment in two cognitive domains, i.e., memory and attention (speed of information processing), which should always be addressed for a comprehensive diagnostic work-up within the spectrum of neurodevelopmental (ICD-11) or neurocognitive disorders (DSM-5).

## Figures and Tables

**Table 1:** Overview of the nine Syndrom-Kurztest (SKT) subtests.

Name of Subtest	Content of Subtest	Domain
I Naming Objects	twelve objects have to be named and memorized at the same time	attention/speed
II Immediate Recall	recall of objects shown in subtest I	memory
III Naming Numerals	two digit numbers written on magnetic blocks placed on a board have to be read out loud	attention/speed
IV Arranging Blocks	the magnetic blocks have to be arranged in ascending order of the numbers	attention/speed
V Replacing Blocks	the blocks have to be replaced in their original positions	attention/speed
VI Counting Symbols	symbols printed on a tableau have to be counted	attention/speed
VII Reversal Naming	two rows composed of two letters in random order are to be read by naming each letter with the name of the other	attention/speed
VIII Delayed Recall	recall of objects shown in subtest I	memory
IX Recognition Memory	identification of objects shown in subtest I from a table containing 48 objects	memory

**Table 2:** Demographic data, Mini-Mental State Examination (MMSE) and depression scores, SKT norm scores for subtests I to IX, SKT subscores for memory and attention and SKT total score. Frequencies (sample size and gender) or mean values with standard deviations in brackets are given.

	MCI M (SD)	DEM M (SD)	DEP M (SD)	*p*	Group Comparisons
sample size	172	166	211		
age	73.9 (7.3)	76.6 (7.6)	72.5 (7.5)	0.000	DEP < MCI < DEM
gender (f/m)	90/82	102/64	134/77	0.045	
education *	12,0 (2,8)	11,3 (2,9)	11,4 (2,8)	*n*.s.	
MMSE **	26.5 (2.2)	22.6 (3.1)	26.2 (3.0)	0.000	DEM < MCI = DEP
depression (z-scores) ***	−0.42 (0.77)	−0.27 (0.81)	0.55 (1.04)	0.000	DEM = MCI, MCI < DEP, DEM < DEP
SKT I	0.70 (0.87)	1.17 (0.90)	0.91 (0.93)	0.000	MCI < (tend.) DEP < DEM
SKT II	0.92 (0.93)	1.64 (0,72)	0.78 (0.93)	0.000	MCI = DEP; DEP < DEM; MCI < DEM
SKT III	0.56 (0.83)	0.89 (0.95)	0.69 (0.90)	0.003	MCI = DEP; DEP = DEM; MCI < DEM
SKT IV	1.01 (0.96)	1.49 (0.84)	1.22 (0.94)	0.000	MCI < (tend.) DEP < DEM
SKT V	1.12 (0.95)	1.50 (0.81)	1.28 (0.91)	0.000	MCI = DEP; DEP < (tend.) DEM; MCI < DEM
SKT VI	0.67 (0.87)	0.99 (0.95)	0.91 (0.96)	0.009	MCI < DEP; DEP = DEM; MCI < DEM
SKT VII	1.06 (0.95)	1.39 (0.89)	1.25 (0.93)	0.005	MCI = DEP; DEP = DEM; MCI < DEM
SKT VIII	1.24 (0.92)	1.77 (0.60)	0.86 (0.94)	0.000	DEP < MCI < DEM
SKT IX	0.87 (0.99)	1.46 (0.89)	0.83 (0.99)	0.000	DEP = MCI; DEP < DEM; MCI < DEM
SKT memory	3.03 (1.85)	4.87 (1.44)	2.47 (2.06)	0.000	DEP < MCI < DEM
SKT attention	5.12 (3.58)	7.42 (3.37)	6.27 (3.95)	0.000	MCI < DEP < DEM
SKT sum score	8.15 (3.97)	12.28 (3.55)	8.74 (4.78)	0.000	MCI= DEP; DEP < DEM; MCI < DEM

Abbreviations: MCI: mild cognitive impairment, DEM: dementia, DEP depression; MMSE: Mini-Mental State Examination, f: female, m: male, tend.: statistical tendency (*p* < 0.10);. * 19 missing; ** 1 missing; *** 53 missing.

**Table 3:** Results of receiver operating curves (ROC): area under curve (AUC).

	MCI	DEM	DEP
SKT sum score	0.83	0.96	0.81
SKT memory subscore	0.77	0.93	0.68
SKT attention subscore	0.74	0.88	0.79
